# Prevalence of HPV infection and cervical lesions among women with pelvic organ prolapse and implications for preoperative evaluation

**DOI:** 10.1186/s12885-025-14049-4

**Published:** 2025-04-07

**Authors:** Yanjun Ge, Fan Wu, Yuchen Zhang, Xintao Wang, Guizhu Wu

**Affiliations:** Shanghai Key Laboratory of Maternal Fetal Medicine, Shanghai Institute of Maternal-Fetal Medicine and Gynecologic Oncology, Shanghai First Maternity and Infant Hospital, School of Medicine, Tongji University, No.2699 Gaokexi Road, Shanghai, 200092 China

**Keywords:** Pelvic organ prolapse, HPV infection, Squamous intraepithelial lesions, Preoperative examination

## Abstract

**Background:**

This study aimed to learn the sketch of HPV infection and cervical lesions including squamous intraepithelial lesions (SIL) and cervical cancer in the population of patients with pelvic organ prolapse and to attempt to provide them with recommendations for preoperative cervical evaluation.

**Methods:**

This was a retrospective analysis of HPV status, cervical cytology, colposcopic results and histopathology in 1292 patients with Pelvic Organ Prolapse (POP) indicated for hysterectomy over a period of more than 5 years.

**Results:**

Of the 1292 patients, 95 (7.4%) patients tested positive for HPV infection. Among these patients with HPV infection, abnormal Thinprep cytologic test (TCT) results were found in 43 (45.3%) patients and abnormal histopathology were found in 43 (45.3%) patients. Among the 40 patients with normal TCT and positive HPV infection (excluding HPV16/18), 3 cases of unexpected LSIL and one case of unexpected HSIL were identified accidentally. Among POP patients with normal TCT and negative HPV results, 6 cases of LSIL and one case of HSIL, as well as 3 cases of grade I endometrioid endometrial adenocarcinoma and 3 cases of complex atypical hyperplasia of endometrium were accidentally detected in postoperative histopathology.

**Conclusions:**

POP patients are not at higher risk of HPV infection but women with POP and positive HPV infection are at higher risk of cytologic abnormalities or cervical lesions than non-POP population. Colposcopy is recommended as part of the preoperative examination of POP patients to rule out cervical lesions, especially those with HPV infection.

## Introduction

Pelvic organ prolapse (POP) is a common health problem among women of advanced age, especially in developing countries. Cervical lesions including squamous intraepithelial lesions (SIL) and cervical cancer also pose a serious threat to elder women. As patients undergoing hysterectomy for pelvic organ prolapse are normally elder women, they may be at a higher risk of developing cervical lesions or other types of gynecological malignancy. It has been reported that the incidence of unanticipated uterine malignancy involving endometrial carcinoma and cervical cancer in cases of hysterectomy for POP was between 0.3% and 0.8% [1–3]. As for unanticipated both premalignant and malignant uterine pathology, the incidence was higher, ranging from 0.94 to 4.2% [1, 3, 4]. For cervical lesions specifically, a meta-analysis showed non-negligible risk of cervical intraepithelial neoplasia or cervical malignancy in hysterectomy specimens performed for POP [5]. One study showed that the risk of cervical intraepithelial neoplasia was 0.78% [1]. Another one showed that the risk of cervical intraepithelial neoplasia and cervical cancer was 1.5%, with 0.3% for sole cervical cancer [3].

Concurrence of POP and cervical cancer is relatively rare but is a complicated and thorny problem. Catarina et al. reported an elderly patient with fourth-degree urogenital prolapse and squamous-cell cervical carcinoma (FIGO stage IIIA) simultaneously, which was difficult to handle [6]. Similarly, Aikaterini-Eirini et al. presented a POP patient diagnosed with stage IIIB cervical cancer, who was offered end-life care measures due to the complicated status [7]. Therefore, it is necessary to pay attention to cervical abnormality of POP patients to prevent progress to concurrence of POP and cervical cancer. As the prerequisite of cervical cancer, the rates of HPV infection and squamous intraepithelial lesion in the population of POP patients are rarely reported. Whether a more rigorous assessment of cervix for POP patients is needed remains a question yet to be answered. In this study we analyzed the HPV status, cervical cytology, colposcopic results and histopathology in patients indicating to hysterectomy for POP, for the purpose of learning the sketch of cervical lesions of POP patients and attempted to provide suggestions for preoperative assessments of cervix for POP patients.

## Materials and methods

This study was approved by the Ethics Committee of Shanghai First Maternity and Infant Hospital and informed consent was signed by the patients before surgery. Patients diagnosed with grade I to IV of pelvic organ prolapse in Shanghai First Maternity and Infant Hospital from Dec.26,2016 to Mar.25,2022 were included in this study. Severity of pelvic organ prolapse was assessed based on Pelvic Organ Prolapse Quantification approved by the International Continence Society [8, 9]. All the included patients were offered cervical cancer screening, namely HPV test and Thinprep cytologic test (TCT). Sampling for HPV and TCT detection was performed by experienced gynecologists. The method for HPV genotyping was the polymerase chain reaction (PCR)-reverse-dot-blot (RDB) HPV genotyping assay, which was performed through DNA extraction, PCR amplification, hybridization, and gene chip analysis on the gene chip reading meter (Hybribio, Guangdong) according to the manufacturer’s instructions. The gene chip can determine 21 HPV subtypes, including 15 high-risk HPV genotypes (HPV16, 18, 31, 33, 35, 39, 45, 51, 52, 53, 56, 58, 59, 66 and 68) and 6 low-risk HPV genotypes (HPV6, 11, 42, 43, 44 and 81).TCT detection results were interpreted by two experienced pathologists separately, and were categorized according to the Bethesda 2001 criteria [10]. The decision of whether to perform colposcopy and loop electrosurgical excision procedure (LEEP) was based on ASCCP guidelines [11, 12]. Specifically, the indications for colposcopy include the following ones: (1) Abnormal cervical cytological screening results, such as LSIL, HSIL, squamous cell carcinoma, atypical glandular epithelial cells, atypical squamous epithelial cells. (2) Abnormal lesions in the cervix are found by visual inspection, such as ulcers or masses. (3) Positive results in visual inspection with acetic acid (VIA) or Lugol’s iodine (VILI). (4) Persistent infection with high-risk HPV. (5) Infection with HPV 16 and/or 18. HPV test, TCT and colposcopy were done within one month before surgery performed for prolapse. Preoperative anesthesia assessment and preoperative laboratory tests including renal and liver functions, complete blood count, coagulation profile, serum electrolyte test, electrocardiography and pulmonary function were routinely offered. Vaginal wash by tincture of iodine was offered before surgery to limit post-operative infection. One’s surgical strategy was decided based on age, health status, type and degree of anatomical defects as well as desired outcomes of the patient. Of all the included 1292 patients, 1228 patients underwent hysterectomy for POP. 64 patients didn’t undergo hysterectomy for private reasons so postoperative histopathology results were not available for those 64 patients. Specimens were sent for histopathological examination after surgery.

We retrospectively analyzed the results of HPV test and TCT of all the included patients, as well as the histopathological findings through colposcopy, after LEEP and after surgery performed for prolapse of patients who underwent corresponding examination/therapy. Information required for analysis were all retrieved from the computerized medical records. Data were collected, tabulated and performed proportion statistics. Results were presented as the number and percentage (%).

## Results

### HPV infection among POP patients

Totally, 1292 patients with pelvic organ prolapse (POP) were included in the study. Baseline characteristics of these patients were shown in Table 1. Firstly, we calculated the rate of HPV infection of these patients and analyzed HPV types and cytology (Table 2). Of the 1292 patients, 95 (7.4%) patients showed HPV infection, of which 23 (24.2%) were positive for HPV16/18, 56 (58.9%) were positive for other high-risk HPV types and 16 (16.8%) were positive for low-risk HPV types. Among the 95 patients with HPV infection, 43 (45.3%) were found with abnormal TCT results and 52 had normal TCT. Notably, among these 52 patients with normal TCT, 12 had HPV 16/18 infection. In addition, 45.3% (43/95) of the patients with HPV infection showed abnormal histopathological results on biopsy under colposcopy, histopathological examination after loop electrosurgical excision procedures (LEEP) or histopathological examination of surgically removed uterus for prolapsed. Specifically, there are 19 patients with HPV infection who had an abnormal histopathological diagnosis based solely on the hysterectomy specimen and 24 who had an abnormal histopathological diagnosis based on biopsy under colposcopy or histopathological examination after LEEP pre-operatively.

**Table 1:** Characteristics of the POP patients included in the study

Variables	Total of 1292 studied women
Age, mean ± SD	64 ± 8.89
Weight (kilogram), mean ± SD	60.08 ± 7.4
Height (meter), mean ± SD	1.59 ± 0.95
Body mass index (BMI), mean ± SD	23.9 ± 2.9
Parity, mean ± SD	1.6 ± 0.81
Number of pregnancies, mean ± SD	2.86 ± 1.24
Number of caesarean sections, mean ± SD	0.06 ± 0.36
Smoking	17(1.3%)
Alcohol use	20(1.5%)
POP-Q* stage	
I	48(3.7%)
II	183(14.2%)
III	1011(78.3%)
IV	50(3.9%)

*POP-Q: Pelvic Organ Prolapse Quantification

**Table 2:** HPV types, cytological and histopathological results among POP patients with HPV infection

HPV status, cytological and histopathological findings	%
positive HPV infection	7.4(95/1292)
HPV16/18	24.2 (23/95)
Other high-risk types	58.9 (56/95)
Low-risk types	16.8 (16/95)
With abnormal TCT findings	45.3 (43/95)
ASC-US	33.7(32/95)
ASC-H	2.1(2/95)
LSIL	7.4(7/95)
HSIL	2.1(2/95)
With abnormal histopathological diagnosis*	45.3 (43/95)
LSIL	25.3(24/95)
HSIL	18.9(18/95)
Complex atypical endometrial hyperplasia	1.1(1/95)

*Including uterine biopsies under colposcopic examination, histopathological examination of uterus after loop electrosurgical excision procedures (LEEP) and histopathological examination of surgically removed uterus for uterovaginal prolapse

### Cytological results among POP patients

We then analyzed the cytological results obtained from TCT. Among the 1292 POP patients, 13.16% (170/1292) had cytological abnormality, including 158 cases of ASC-US, 9 cases of LSIL and 3 cases of HSIL. Among the 95 patients with positive HPV infection, 45.3% (43/95) had abnormal TCT findings, including 32 cases of ASC-US, 2 cases of ASC-H, 7 cases of LSIL and 2 cases of HSIL.

Notably, of the 158 patients with ASC-US, 18.4% (29/158) were diagnosed with histopathological abnormality by biopsy under colposcopy and/or postoperative histopathology (specifically, 20 cases of LSIL, 8 cases of HSIL and 1 case of cervical adenocarcinoma in situ). 21.5% (34/158) were positive for HPV, of which 52.9% were diagnosed with histopathological abnormality.

### Colposcopy results among POP patients

Of all the cases involved, 59 cases needed colposcopy according to guidelines, while only 48 patients actually underwent colposcopy and 31 patients had abnormal colposcopic results (Table 3). Notably, among these 48 patients, 21 patients showed a lower grade of postoperative histopathological lesions compared to colposcopic results, 11 patients showed a higher grade of postoperative histopathological lesions compared with colposcopic results, and 10 patients showed the identical grade of postoperative histopathological lesions as colposcopic results (the other 6 patients didn’t undergo surgery for private reasons).

There are 124 patients with ASCUS and no HPV infection and 40 patients with normal TCT results and positive for HPV excluding HPV 16/18. These 164 patients didn’t require colposcopy according to the guidelines but 38 out of them underwent colposcopy. Interestingly, 12 patients showed abnormality colposcopic results. Besides, 12 of these 38 patients showed histopathological lesions after LEEP or surgery.

**Table 3:** Colposcopic results of 48 POP patients who needed colposcopy according to guidelines

Colposcopic results	Number of patients
LSIL	17(35.4%)
HSIL	14(29.2%)
Chronic cervicitis	17(35.4%)

### Accidental detection of cervical lesions among POP patients with HPV infection

**Fig. 1:**
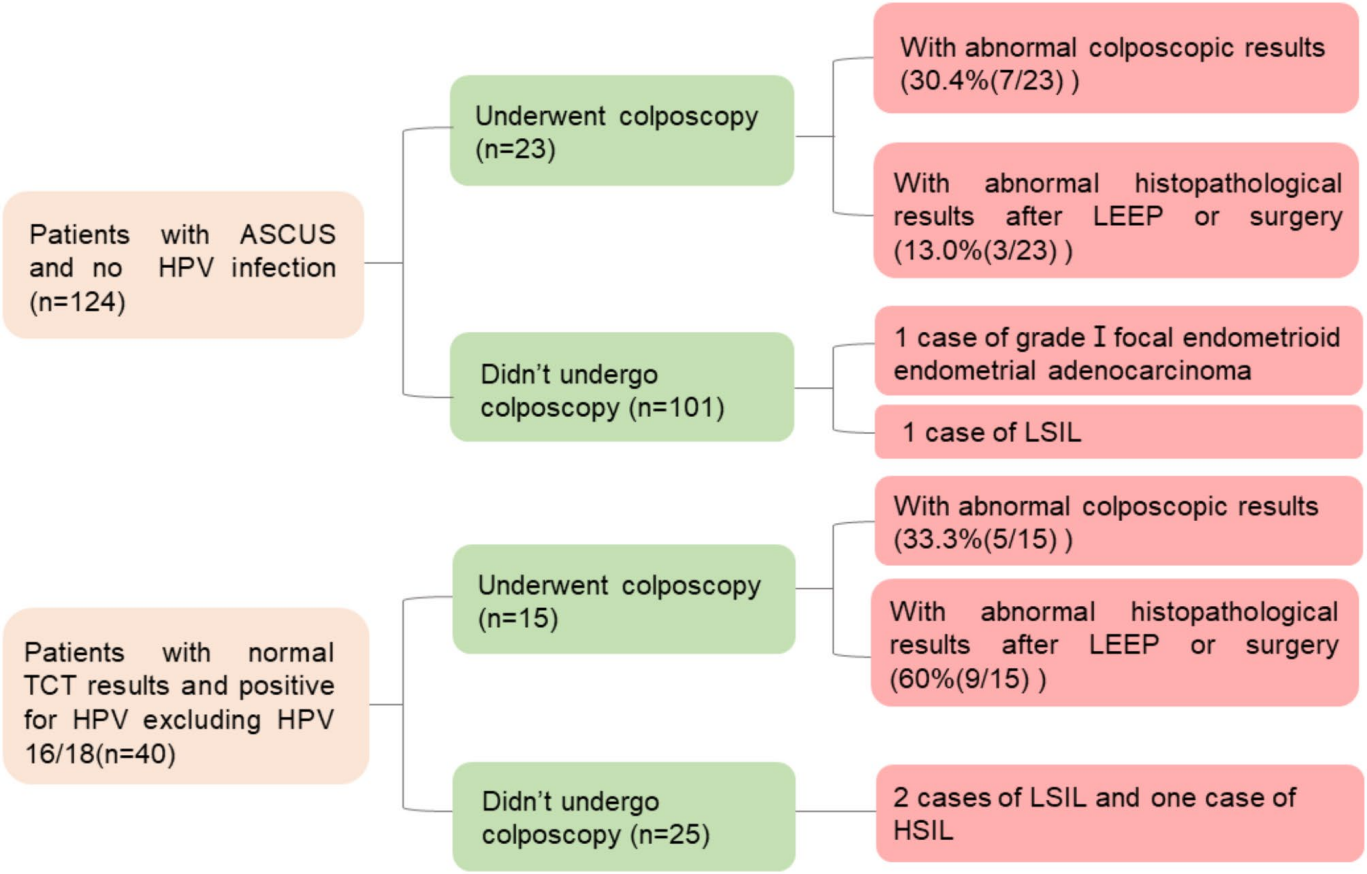
POP patients who didn’t need colposcopy according to guidelines were accidentally found with some colposcopic or histopathological abnormality

Among the 164 patients mentioned above (124 patients with ASCUS and no HPV infection and 40 patients with normal TCT results and positive for HPV excluding HPV 16/18), 126 patients didn’t undergo colposcopy. Among these 126 patients, 4 cases of LSIL, 1 case of HSIL and 1 case of grade I focal endometrioid endometrial adenocarcinoma were accidentally found. Among the 126 patients, 25 had normal TCT and were infected with HPV excluding HPV16/18, and 3 cases of unexpected LSIL and one case of unexpected HSIL were among them. Unexpected colposcopic or histopathological abnormality in POP patients who were not required to receive colposcopy according to guidelines are showed in Fig. 1.

### Accidental detection of cervical lesions among POP patients with normal TCT/HPV

Among patients who had normal TCT and HPV testing results and thereby didn’t undergo colposcopy, 6 cases of LSIL and one case of HSIL were unexpectedly detected through histopathological examination after uterine prolapse surgery. Notably, 3 cases of grade I endometrioid endometrial adenocarcinoma and 3 cases of complex atypical hyperplasia of endometrium were accidentally identified.

## Discussion

Cervical cancer and pelvic organ prolapse are both non-negligible threats to elder women [13, 14]. Whether pelvic organ prolapse has influence on the occurrence of cervical lesions and the results of cervical examination remains a question. Here we included 1292 POP patients and analyzed their HPV status, cervical cytology, colposcopic results and histopathology after hysterectomy.

Our analysis showed that the HPV infection rate in POP patients was 7.4%, which is similar to or lower than that of non-POP population according to data from different districts in China [15–17]. Therefore, POP may not increase the risk of HPV infection. A recent study reported that the rate of high-risk HPV infection in POP patients was 10.3% and is not higher than non-POP population [18]. The results were similar to ours. Of all the included POP patients positive for HPV, the majority (83.2%) were infected with high-risk HPV types (Table 2). Notably, we found that nearly 50% of the patients with HPV-infected POP had cytologic abnormalities and nearly 50% of those were eventually diagnosed with cervical lesions according to histopathological results. Thus, POP patients are not at higher risk of HPV infection than non-POP population but women with POP and positive HPV infection are at higher risk of cytologic abnormalities or cervical lesions. HPV persistence is one of the most significant factors predicting the risk of developing recurrent high-grade cervical dysplasia [19]. Therefore, the result prompted us to pay more attention to the management of HPV infection in POP patients. Routine HPV test is of great significance to cervical cancer screening, and for POP patients it is necessary to pay more attention when HPV screening results are positive. HPV vaccine is an effective way to protect against HPV infection and squamous intraepithelial lesion for POP patients. One study has even demonstrated that HPV vaccination would protect against lower genital tract dysplasia in the absence of the uterine cervix for patients having underwent hysterectomy [20].

Forty-eight patients underwent cervical biopsy upon colposcopy according to guidelines, and we compared their colposcopic diagnosis and cervical pathological results of surgical specimen. We found that nearly half (21/48) were found with “pathological downgrading”. This result points out that patients with pelvic organ prolapse may be easily mistaken for lesions upon colposcopy. In some cases, colposcopy may be difficult for POP patients considering that decubitus and ulceration of the prolapsed cervix is common in POP patients. Therefore it warrants further study of colposcopic diagnosis in POP patients.

We offered colposcopic cervical biopsies to 23 POP patients with ASC-US and HPV-negative and 15 POP patients with normal cytology and HPV infection (excluding HPV 16/18). Totally 12 cases of cervical lesions were detected, which is a high rate of 31.8%. In addition, among 126 patients who didn’t need colposcopy but actually received the examination, 3 cases of unexpected LSIL and one case of unexpected HSIL were identified. These four patients all had normal TCT but positive HPV infection. Therefore, we recommend strengthened managements for POP patients with HPV infection and the indication for colposcopy could be expanded considering the low invasiveness and accessibility of the examination.

We also explored the risk of accidental endometrial lesion and endometrial carcinoma in POP patients. Among the POP patients receiving hysterectomy who were negative for both HPV and TCT, 6 cases of LSIL and one case of HSIL were accidentally diagnosed; however, 6 cases were unexpectedly diagnosed with more severe endometrial lesions, including 3 cases of grade I endometrioid endometrial adenocarcinoma and 3 cases of complex atypical hyperplasia of endometrium. In addition to these 6 cases, there was one patient who was found with ASC-US and grade I endometrioid endometrial adenocarcinoma accidentally in postoperative histopathology. Therefore, totally 7 patients (0.54%) were accidentally found with endometrial lesions on postoperative histopathological examination. The proportion is close to or even lower than literature reports (0.94-2.7%) [1, 3], which indicates our adequate assessment for the risk of endometrial lesions prior to surgery in POP patients. Routinely, for patients with premenopausal menstrual disorders or postmenopausal bleeding or with an endometrium of 5 mm or more detected through ultrasonography, hysteroscopic endometrial biopsy was performed to fully assess the risk of endometrial lesions. In addition, the risk of accidental cervical lesions is 0.54% in the current study, which is lower than literature reports and most are merely LSIL [3]. This may be due to the high percentage of colposcopy performed on POP patients in our hospital (6.6% of 1292 POP patients indicated to hysterectomy and 81.4% of 59 POP patients requiring colposcopy according to guidelines), which leads to a comprehensive and adequate cervical assessment. Therefore, colposcopy is recommended as part of the preoperative examination of POP patients to rule out cervical lesions, especially those with HPV infection.

## Conclusions

POP patients with HPV infection are at a high risk of cervical lesions. Therefore, management should be strengthened and it can be considered that all the POP patients with HPV infection should undergo colposcopy examination. As rigorous assessment of cervix can reduce the risk of accidental cervical lesions detected in postoperative histopathology in POP patients, uterus-sparing treatment can be applied to those who have underwent adequate assessments of cervix and desire to preserve the uterus.

## Data Availability

No datasets were generated or analysed during the current study.

## Abbreviations

POP: Pelvic organ prolapse
TCT: Thinprep cytologic test
HPV: Human papillomavirus
HSIL: High-grade squamous intraepithelial lesion
LSIL: Low-grade squamous intraepithelial lesion
LEEP: Loop electrosurgical excision procedures
ASC-US: Atypical squamous cell of undetermined significance
